# Comparative Performance of Artificial Intelligence‐Based Computer‐Aided Detection Systems for Colorectal Polyps: A Systematic Review and Network Meta‐Analysis

**DOI:** 10.1111/den.70138

**Published:** 2026-03-29

**Authors:** Satoshi Shinozaki, Jun Watanabe, Takeshi Kanno, Katsuyuki Nakazawa, Tomonori Yano

**Affiliations:** ^1^ Shinozaki Medical Clinic Utsunomiya Tochigi Japan; ^2^ Division of Gastroenterology, Department of Medicine Jichi Medical University Shimotsuke Japan; ^3^ Division of Gastroenterological, General and Transplant Surgery, Department of Surgery Jichi Medical University Shimotsuke Tochigi Japan; ^4^ Division of Community and Family Medicine Jichi Medical University Shimotsuke Tochigi Japan; ^5^ Division of Gastroenterology and Farncombe Family Digestive Health Research Institute, Department of Medicine McMaster University Hamilton Ontario Canada; ^6^ R & D Division of Career Education for Medical Professionals, Medical Education Center Jichi Medical University Shimotsuke Tochigi Japan; ^7^ Division of Gastroenterology Tohoku University Graduate School of Medicine Sendai Miyagi Japan; ^8^ Japan Community Health Care Organization Utsunomiya Hospital Utsunomiya Tochigi Japan

**Keywords:** artificial intelligence, colonoscopy, colorectal polyp, computer‐aided detection, endoscopy

## Abstract

**Background and Aims:**

Computer‐aided detection (CADe) is anticipated to enhance adenoma detection rate (ADRs). The aim of this study was to systematically collect randomized‐controlled trials comparing colonoscopy with CADe to standard colonoscopy without CADe in ADRs.

**Methods:**

We performed a Bayesian network meta‐analysis of randomized‐controlled trials. Three electronic databases including MEDLINE, Embase, and the Cochrane Central Register of Controlled Trials were searched. The primary outcome was the comparison of the performance of CADe systems in ADRs; the secondary outcome was the sessile serrated lesions detection rates (SSLDRs).

**Results:**

A total of 48 randomized controlled trials involving 38,986 patients were included in the quantitative analysis. Several CADe systems improved ADR compared with controls that ENDO‐AID (risk ratio [RR] 1.26, 95% credible interval [CrI] 1.14–1.40), CADEYE (RR 1.18, 95% CrI 1.10–1.26), and GI Genius (RR 1.15, 95% CrI 1.08–1.22) were supported by moderate confidence evidence according to the Confidence in Network Meta‐Analysis (CINeMA). For SSLDR, ENDO‐AID (RR 1.36, 95% CrI 1.03–1.79) and GI Genius (RR 1.25, 95% CrI 1.08–1.46) may offer improved detection compared with controls. Across multiple sensitivity analyses excluding studies by withdrawal time, conflicts of interest, limited study numbers, image‐enhanced endoscopy, non‐parallel design, single‐center settings, operator experience, or earlier publication years, the direction and magnitude of ADR improvements with CADe systems remained largely consistent with the primary analysis.

**Conclusions:**

Based on the CINeMA framework, the certainty of evidence ranged from low to moderate, indicating that some CADe systems are likely to improve ADR.

## 1 Introduction

Adenoma detection rate (ADR) during colonoscopy is a reliable benchmark for quality control, and a 1% improvement of ADR results in a 3% decrease in interval cancers [1]. A large European study reported that more than 20% ADR significantly decreased interval cancers after screening colonoscopy [2]. The serrated pathway is believed to account for approximately 15%–20% of colorectal cancers, producing tumors that differ from conventional colorectal cancers in their clinical presentation, morphological features, and underlying molecular alterations [3]. A recent US study demonstrated that an improvement of sessile serrated lesion detection rates (SSLDR) results in a significant decrease in interval cancer [4]. The Japan polyp study cohort showed that endoscopic colorectal polyp resection decreased colorectal cancer by 86% [5]. Although the improvement of ADR leads to future reduction of colorectal cancer incidence, the miss rate of adenoma cannot be zero even in experienced endoscopists. A systematic review calculated miss rates of 26% for adenoma and 27% for serrated lesions based on the tandem colonoscopy studies [6]. Methods to prevent missed adenomas and sessile serrated lesions (SSLs), thereby improving ADR and SSLDR, have been attracting attention.

The development and dissemination of computer‐aided detection (CADe) systems utilizing artificial intelligence (AI) improves ADR by 28% [7]. The use of CADe may reduce medical costs as well as colorectal cancer incidence reduction [8]. Recent systematic reviews evaluating several CADe systems demonstrated the usefulness of CADe for the improvement of ADR [9, 10]. However, these mixed results including various kinds of CADe systems based on different AI‐platforms had the inherent heterogeneity across randomized‐controlled trials (RCTs). Despite the growing body of CADe research on ADRs, there are no network meta‐analyses comparing ADRs or SSLDRs among CADe systems.

This study aimed to systematically collect RCTs comparing colonoscopy with CADe versus standard colonoscopy without CADe, and to evaluate which CADe system is most effective.

## 2 Methods

### 2.1 Registration

We performed a systematic review and Bayesian network meta‐analysis of RCTs comparing CADe systems with standard colonoscopy. This study was conducted according to the Preferred Reporting Items for Systematic Review and Meta‐analysis (PRISMA) and to the PRISMA for Network Meta‐Analyses (Appendix S1) [11, 12]. This study protocol was registered in the International Prospective Register of Systematic Reviews (PROSPERO Registration ID: CRD420251129376).

### 2.2 Eligibility Criteria

In this review, CADe refers to AI‐based systems designed to assist endoscopists in the real‐time identification of colorectal polyps during colonoscopy. These systems analyze endoscopic video streams and generate visual or auditory alerts when potential polyps are detected. Patients with colorectal polyps evaluated by CADe‐assisted colonoscopy and standard colonoscopy were included. The primary outcome was to compare the efficacy of CADe‐assisted colonoscopy in improving the ADR compared to standard colonoscopy across different CADe systems. The secondary outcome was to compare the detection rate of SSLDR using CADe‐assisted colonoscopy versus standard colonoscopy in a similar manner across the various CADe systems.

The inclusion criteria of the network meta‐analysis were as follows: (1) full‐length, peer‐reviewed RCTs; (2) ADRs were reported or could be calculated; and (3) comparison between CADe‐assisted colonoscopy and standard colonoscopy was evaluated. We excluded the following studies: (1) patients under 15 years old; (2) mixed data from two or more CADe systems, and the data cannot be divided into each device; (3) studies that did not report critical data; (4) retrospective studies, prospective observational studies, review articles, or case reports; (5) no description of the name of CADe devices; (6) Gray literature, including conference abstracts, trial registry records without full publications, letters, editorials, and review articles. Any clinical indications were included.

### 2.3 Search Strategy

We conducted a comprehensive bibliographic search of the following databases from 2015 to August 16, 2025: MEDLINE (Ovid), Embase (Ovid), and the Cochrane Central Register of Controlled Trials (CENTRAL) (Ovid). We searched for text words as well as controlled vocabulary (MeSH or Emtree terms) related to colonic polyps or adenomas, colonoscopy or endoscopy, and computer‐aided detection (CADe) systems, using Boolean operators (AND, OR, NOT). Synonyms, related terms, and variations of root words were also searched. The search strategy was developed in MEDLINE and translated to Embase and CENTRAL with database‐specific adaptations. The starting year of 2015 was chosen to reflect the emergence and clinical implementation of AI‐based CADe systems in gastrointestinal endoscopy [13]. A recursive manual search of the bibliographies of eligible studies and relevant systematic reviews was conducted to identify additional articles. Furthermore, experts in the field were contacted to ensure the inclusion of all relevant studies. No language restrictions were applied to the literature search. Studies published in any language were considered eligible, provided that sufficient data could be extracted for quantitative synthesis. The detailed search strategy is included in Appendix S2.

We also searched the guidelines of the European Society of Gastrointestinal Endoscopy, the American Society for Gastrointestinal Endoscopy, and the Japan Gastroenterological Endoscopy Society, as well as the World Health Organization International Clinical Trials Platform Search Portal and ClinicalTrials.gov, to identify ongoing or unpublished studies.

### 2.4 Study Selection

Two reviewers (S.S. and J.W.) independently conducted a search and screened the titles and abstracts of all retrieved records. Full texts of potentially eligible studies were then reviewed independently according to predefined inclusion and exclusion criteria. Studies that were identified as duplicates were removed. Data extraction was initially performed by one author (S.S.), and the accuracy of the extracted data was confirmed by another author (J.W.). Any disagreements during the selection or extraction phases were resolved through discussion. If a consensus was not reached, a third reviewer (T.K.) provided a final decision.

### 2.5 Data Extraction and Quality Assessment

We extracted the following data from published articles: year of publication, country, study design, type of CADe system, indication, number of centers, number of patients, age, and number of endoscopists. When essential data were unclear or missing, we contacted the corresponding authors directly to obtain further details. In cases where tandem colonoscopy studies were included, we extracted data from the initial (first pass) examinations to ensure consistency across trials and to reduce the Risk of Bias related to repeated inspection.

### 2.6 Risk of Bias

The first and second authors independently evaluated the Risk of Bias and applicability using the Risk of Bias 2 (RoB 2) at the outcome measure level [14]. The Risk of Bias tool assesses the Risk of Bias in five domains: randomization process, deviations from intended interventions, missing outcome data, measurement of the outcome, and selection of the reported result. The Risk of Bias was categorized as high risk, low risk, and some concerns. Two reviewers (S.S. and J.W.) piloted the tool on a sample of studies to ensure consistency in interpretation. In case of disagreements between two authors, the third author (T.K.) was involved in reaching a consensus.

### 2.7 Data Synthesis and Statistical Analysis

We calculated the relative risk ratios (RRs) and 95% credible intervals (CrIs) for ADR and SSLDR. For dichotomous outcomes and group‐level data, a binomial likelihood approach was applied. Study effect sizes were pooled using a Bayesian network meta‐analysis framework with a random‐effects model and non‐informative normal priors. To address the dependencies introduced by multi‐arm trials, multivariate distributions were used. The heterogeneity variance in the random‐effects model reflected both within‐comparison and between‐study variability.

As a supplementary analysis to enhance clinical interpretability by presenting absolute effects, we performed an additional network meta‐analysis using risk difference (RD) as the effect measure. This analysis was intended to complement (rather than replace) the primary Bayesian RR‐based network meta‐analysis and to assess the robustness of conclusions across effect measures and estimation frameworks. This RD‐based network meta‐analysis was conducted within a frequentist framework using a random‐effects model to leverage a well‐established and numerically stable estimation approach for RD in network meta‐analysis. Absolute differences in ADR between each CADe device and control were estimated, together with corresponding 95% confidence intervals (CIs), using the same evidence network meta‐analysis and modeling assumptions.

We converted RR estimates for CADe systems with high confidence into absolute effects using three representative baseline ADR scenarios informed by external data: 25% (average‐risk screening colonoscopy), 35% (post‐polypectomy surveillance), and 50% (FIT‐positive colonoscopy). These values were chosen based on population‐based registry and benchmark ADR data [15, 16] and rounded for clinical interpretability. For each scenario, the absolute increase in ADR was calculated as baseline risk × (RR−1), and the number needed to screen (NNS) was defined as the reciprocal of this absolute risk difference.

We additionally performed pairwise meta‐analyses for selected CADe systems to examine within‐device consistency and between‐study variability. For each CADe system, randomized controlled trials directly comparing the specific CADe device with control were included. Pooled RRs and 95% CIs were calculated using both random‐effects and fixed‐effect models, with the random‐effects model considered primary due to anticipated clinical and methodological heterogeneity across studies. Effect estimates were displayed on a logarithmic scale. These pairwise analyses were conducted as supplementary analyses to support and contextualize the findings of the network meta‐analysis.

Treatment rankings for each outcome were assessed using the surface under the cumulative ranking curve (SUCRA) and average ranks [17]. We also employed the MetaInsight platform for analysis [18]. By comparing the distributions of the effect modifiers, country, age, and experience of endoscopists, we evaluated transitivity across the different comparisons [19]. Incoherence was examined by evaluating the mean estimates, confidence intervals, and Bayesian *p*‐values for direct, indirect, and network‐derived comparisons [18]. Since the network did not include closed loops, formal statistical inconsistency tests were not feasible. In accordance with PRISMA‐NMA and the Confidence in Network Meta‐Analysis (CINeMA) guidance, coherence was assessed through evaluation of transitivity and global model fit.

Two independent reviewers (S.S. and J.W.) assessed the confidence in the evidence for each outcome using the CINeMA tool [20]. Discrepancies in judgment were resolved through discussion, with a third reviewer (T.K.) providing arbitration when needed. The CINeMA approach evaluates six key domains: within‐study bias, across‐studies bias, indirectness, imprecision, heterogeneity, and incoherence. For within‐study bias and indirectness, CINeMA determines the contribution of each study to the overall network estimates and integrates these weights with study‐level assessments to rate the certainty of the evidence (very low, low, moderate, or high) for each treatment comparison.

### 2.8 Sensitivity Analysis

To assess the robustness of the findings and the validity of the conclusions of this review, we conducted a series of sensitivity analyses addressing potential sources of bias, heterogeneity, and effect modification.

First, to minimize the influence of procedural factors, analyses were restricted to studies reporting no statistically significant difference in withdrawal time between groups. In addition, to evaluate the potential impact of conflicts of interest (COI), we performed a sensitivity analysis excluding studies in which at least one author had a relationship with CADe manufacturing companies, including joint development or coauthorship with industry‐affiliated investigators, research funding or sponsorship, lecture honoraria, provision of CADe equipment, or free or loaned use of CADe systems.

To examine the robustness of the network estimates against sparse evidence and methodological variability, further sensitivity analyses were conducted by restricting the analysis to CADe systems supported by three or more RCTs, excluding studies in which CADe was evaluated in combination with image‐enhanced endoscopy (IEE) rather than conventional white‐light imaging, and limiting the analysis to trials with a parallel‐group design.

Additional sensitivity analyses explored the influence of study setting, operator expertise, and publication period by restricting the analysis to multicenter trials, studies in which colonoscopies were performed exclusively by experienced endoscopists (as defined in each original study), and RCTs published between 2023 and 2025, respectively.

All sensitivity analyses were performed using the same Bayesian random‐effects network meta‐analysis framework as in the primary analysis, with RRs and 95% CrIs estimated. All other methodological assumptions and analytical procedures were identical to those of the main analysis.

## 3 Results

### 3.1 Study Selection

**FIGURE 1:**
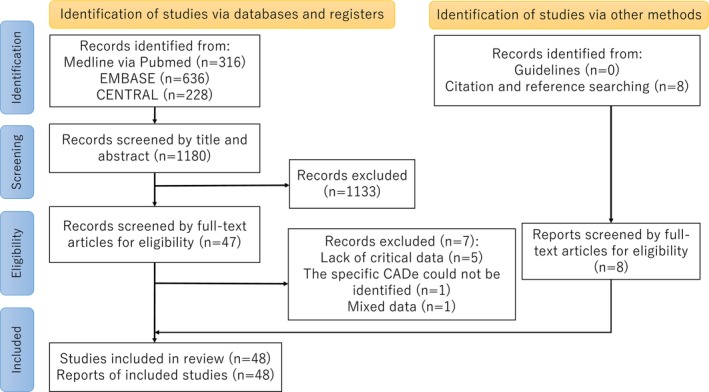
Flow diagram of the study selection process. CADe, Computer‐aided detection.

The study selection process is detailed in Figure 1. As of August 16, 2025, 1180 records were identified from databases and registers. After reviewing titles and abstracts, 1133 records were excluded, leaving 47 records for full‐text screening. Following the screening, seven additional records were excluded. Citation and reference searching identified eight additional articles. In total, 48 studies involving 38,986 patients were included in the quantitative analysis [21, 22, 23, 24, 25, 26, 27, 28, 29, 30, 31, 32, 33, 34, 35, 36, 37, 38, 39, 40, 41, 42, 43, 44, 45, 46, 47, 48, 49, 50, 51, 52, 53, 54, 55, 56, 57, 58, 59, 60, 61, 62, 63, 64, 65, 66, 67, 68].

### 3.2 Characteristics of Studies

Among the 48 RCTs (Table 1), a total of 16 distinct CADe systems were evaluated. CADEYE (12 trials), GI Genius (11 trials), EndoScreener (5 trials), ENDO‐AID (5 trials), and EndoAngel (3 trials) constituted the major evidence base, while the remaining CADe systems were each evaluated in one or two RCTs. The publication year ranged from 2019 to 2025. Of the 48 studies, seven were tandem and 41 were parallel in design. Twenty‐two studies were conducted as multicenter trials, and 23 were performed exclusively by experienced endoscopists. In all tandem studies included, first‐pass data were obtained for analysis. The assessment of transitivity across these studies regarding country, age, and experience of endoscopists was presented in the Appendix S3. Overall, there was no evidence of concern regarding transitivity across the comparisons. Since the network did not contain closed loops, formal statistical assessment of incoherence based on direct and indirect comparisons was not feasible. Therefore, incoherence was evaluated primarily through assessment of transitivity and overall model fit, and no major concerns were identified. The indications for colonoscopy in each study are summarized in Appendix S4.

**TABLE 1:** Characteristics of 48 studies included in this network meta‐analysis.

First author	CADe system	Year	Country	Design	Centers, *n*	CADe, ADR (*n*)	Control, ADR (*n*)
Alali [21]	CADEYE	2025	Kuwait	Parallel	1	48% (24/51)	38% (19/51)
Aniwan [22]	CADEYE	2023	Thailand	Parallel	1	53% (163/312)	42% (130/310)
Desai [23]	CADEYE	2024	United States	Parallel	12	47% (238/509)	43% (224/522)
Djinbachian [24]	CADEYE	2025	Canada	Parallel	1	50% (113/229)	39% (91/238)
Hiratsuka [25]	CADEYE	2025	Japan	Tandem	1	75% (36/48)	72% (33/46)
Hüneburg [26]	CADEYE	2023	Germany	Parallel	1	36% (18/50)	27% (12/46)
Miyaguchi [27]	CADEYE	2024	Japan	Parallel	1	59% (235/400)	44% (174/400)
Nakashima [28]	CADEYE	2023	Japan	Parallel	1	60% (123/207)	48% (99/208)
Rondonotti [29]	CADEYE	2022	Italy	Parallel	5	54% (217/405)	46% (179/395)
Tiankanon [30]	CADEYE	2024	Thailand	Parallel	5	50% (200/400)	39% (153/400)
Yamaguchi [31]	CADEYE	2024	Japan	Parallel	3	59% (66/113)	61% (72/118)
Zimmermann‐Fraedrich [32]	CADEYE	2025	Germany	Parallel	12	41% (330/812)	39% (312/815)
Ahmad [33]	GI Genius	2023	Germany	Parallel	1	72% (220/308)	65% (199/306)
Engelke [34]	GI Genius	2023	Germany	Parallel	1	34% (41/122)	19% (20/110)
Karsenti [35]	GI Genius	2023	France	Parallel	1	38% (376/1003)	34% (341/1012)
Lagström [36]	GI Genius	2025	Denmark	Parallel	4	59% (236/400)	47% (184/395)
Mangas‐Sanjuan [37]	GI Genius	2023	Spain	Parallel	6	65% (1033/1610)	62% (990/1603)
Ortiz [38]	GI Genius	2024	Spain, Italy, Germany, Belgium	Parallel	17	33% (70/214)	37% (79/216)
Repici, *Gut* [39]	GI Genius	2022	Italy	Parallel	10	54% (176/330)	45% (147/330)
Repici, *Gastro* [40]	GI Genius	2020	Italy	Parallel	3	55% (187/341)	41% (139/344)
Seager [41]	GI Genius	2024	United Kingdom	Parallel	12	57% (555/980)	49% (477/986)
Thiruvengadam [42]	GI Genius	2024	United States	Parallel	1	43% (234/550)	35% (189/550)
Wallace [43]	GI Genius	2022	United Kingdom, United States, Italy	Tandem	8	63% (72/116)	62% (70/114)
Glissen Brown [44]	EndoScreener	2022	United States	Tandem	4	51% (57/113)	44% (48/110)
Liu, *Ther Adv* [45]	EndoScreener	2020	China	Parallel	1	29% (114/393)	21% (83/397)
Wang, *Gastro* [46]	EndoScreener	2020	China	Tandem	1	35% (64/184)	27% (49/185)
Wang, *Lancet* [47]	EndoScreener	2020	China	Parallel	1	35% (165/484)	28% (134/478)
Wang, GR [48]	EndoScreener	2023	China	Parallel	4	26% (164/636)	24% (150/625)
Gimeno‐Garcia [49]	ENDO‐AID	2023	Spain	Parallel	1	57% (88/155)	45% (70/157)
Lui [50]	ENDO‐AID	2024	Hong Kong	Parallel	1	54% (128/238)	47% (99/214)
Lau [51]	ENDO‐AID	2024	Hong Kong	Parallel	1	58% (222/386)	45% (169/380)
Spada [52]	ENDO‐AID	2025	Italy	Parallel	2	51% (290/578)	41% (235/580)
Vilkoite [53]	ENDO‐AID	2023	Latvia	Parallel	1	31% (59/194)	21% (43/206)
Gong [54]	EndoAngel	2020	China	Parallel	1	17% (58/355)	8% (27/349)
Yao, *Endosc* [55]	EndoAngel	2022	China	Parallel	1	22% (57/268)	15% (40/271)
Yao, GIE [56]	EndoAngel	2024	China	Tandem	3	21% (47/227)	21% (48/229)
Wei, AJG [57]	EndoVigilant	2023	United States	Parallel	4	36% (139/387)	38% (142/382)
Wei, AIMI [58]	EndoVigilant	2023	United States	Parallel	1	69% (85/124)	80% (96/120)
Kamba [59]	Modified YOLOv3 (LPIXEL)	2021	Japan	Tandem	4	65% (111/172)	54% (93/174)
Lachter [60]	DEEP	2023	Israel	Parallel	1	37% (122/330)	27% (93/344)
Liu, Saudi [61]	Henan Xuanweitang Medical	2020	China	Parallel	1	40% (199/508)	24% (124/518)
Maas, *Endosc* [62]	DISCOVERY	2024	Europe, Canada	Parallel	7	39% (96/250)	38% (93/247)
Shaukat [63]	SKOUT	2022	United States	Parallel	5	48% (326/682)	44% (297/677)
Su [64]	AQCS	2020	China	Parallel	1	29% (89/308)	17% (52/315)
Wang, *Gut* [65]	SegNet	2019	China	Parallel	1	30% (152/522)	21% (109/536)
Xu [66]	Eagle‐Eye	2023	China	Parallel	6	40% (606/1519)	33% (499/1540)
Maas, *Lancet* [67]	MAGENTIQ‐COLO	2024	Europe, United States, Israel	Tandem	10	38% (167/449)	30% (138/467)
Yabuuchi [68]	EndoBRAIN‐EYE	2025	Japan	Parallel	1	51% (254/501)	55% (271/497)

Abbreviations: ADR, adenoma detection rate; AIMI, *Artif Intell Med Imaging*; AJG, *American J Gastroenterol*; CADe, computer‐aided detection; GIE, *Gastrointest Endosc*; GR, *Gastroenterol Rep*.

### 3.3 Certainty of Evidence (CINeMA Assessment)

Following the inclusion of a total of 48 RCTs, the network meta‐analysis and CINeMA assessments were comprehensively evaluated (Appendix S5). According to the CINeMA framework, the certainty of evidence for ADR varied across CADe systems (*n* = 16). High confidence evidence supported the effects of CADEYE, GI Genius, and ENDO‐AID, whereas the certainty for other systems ranged from moderate to low. For some CADe systems, the certainty of evidence was downgraded primarily due to imprecision, as effect estimates were not fully consistent across multiple sensitivity analyses. In contrast, no major concerns were identified regarding indirectness or incoherence. Additionally, CADe systems evaluated in fewer than three RCTs were further downgraded for imprecision and within‐study bias, reflecting the limited amount of direct evidence and the resulting uncertainty in network estimates.

Assessment of publication bias and small‐study effects is summarized in Appendix S6. Visual inspection of comparison‐adjusted funnel plots did not reveal marked asymmetry. In addition, linear regression‐based tests showed no statistically significant evidence of small‐study effects.

### 3.4 Comparison of ADRs Across 16 CADe Devices

**FIGURE 2:**
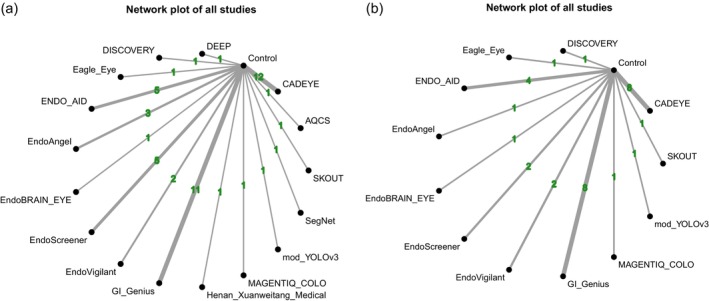
Network plots of all included studies comparing computer‐aided detection (CADe) devices with control in terms of adenoma detection rates (ADRs) (a) and sessile serrated lesion detection rates (SSLDRs) (b).

**FIGURE 3:**
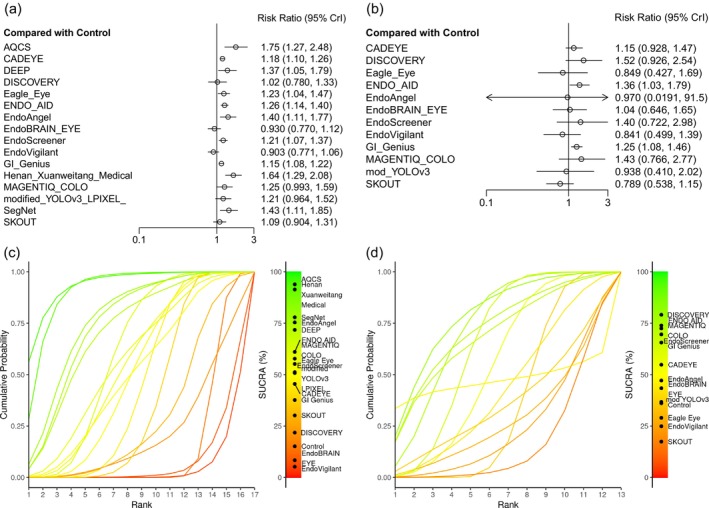
Forest plots comparing computer‐aided detection (CADe) devices with control in terms of adenoma detection rates (ADRs) (a) and in sessile serrated lesions detection rates (SSLDRs) (b). SUCRA (Surface Under the Cumulative Ranking Curve)‐based rankograms (c, d): Higher SUCRA values and curves located closer to the top‐left corner indicate better performance in ADRs (c) and in SSLDRs (d) [69].

The network plot comparing CADe systems with controls in terms of ADRs is presented in Figure 2a. As illustrated in Figure 3a, 10 of the 16 CADe devices showed statistically significant improvements in ADR compared with control. Nevertheless, the extensive overlap of the corresponding 95% CrIs suggests that there were no clear differences in ADR performance among these devices. The SUCRA‐based rankings are presented in Figure 3c and Appendix S7. Among them, CADEYE, ENDO‐AID, and GI Genius exhibited narrower credible intervals, indicating greater precision of the estimated effects, and were rated as having high confidence evidence in the CINeMA framework.

In absolute terms, the risk difference‐based analysis (RD‐NMA) showed an increase in ADR of approximately 5–10 percentage points, depending on the CADe system (Appendix S8). For the three CADe systems supported by high‐confidence evidence in CINeMA (ENDO‐AID, CADEYE, GI Genius), translation of RRs into absolute terms suggested clinically relevant gains across a range of baseline ADRs (Table 2). For example, assuming a screening baseline ADR of 25%, ENDO‐AID would increase ADR to approximately 31.5% (65 more patients with at least one adenoma detected per 1000 colonoscopies; NNS 16), while CADEYE and GI Genius would yield 45 and 37 additional detections per 1000, respectively, corresponding to NNS values of 23 and 27. Similar or larger absolute gains were observed in surveillance and FIT‐positive‐like scenarios.

**TABLE 2:** Estimated absolute ADR gains and number needed to screen (NNS) (95% CrI) for all CADe with statistically significant ADR improvement.

Risk category	Baseline ADR	ADR with CADe^a^	Risk difference	NNS^b^	Confidence rating
AQCS (RR 1.75, 95% CrI 1.27–2.48)					
Low (screening, 25%)	250 per 1000	438 per 1000 (318–620)	188 (68–370)	6 (3–15)	Low
Moderate (surveillance, 35%)	350 per 1000	612 per 1000 (444–868)	262 (94–518)	4 (2–11)	
High (FIT‐positive–like, 50%)	500 per 1000	875 per 1000 (635–1240)	375 (135–740)	3 (1–8)	
CADEYE (RR 1.18, 95% CrI 1.10–1.26)					
Low	250 per 1000	295 per 1000 (275–315)	45 (25–65)	23 (15–40)	High
Moderate	350 per 1000	413 per 1000 (385–441)	63 (35–91)	16 (11–29)	
High	500 per 1000	590 per 1000 (550–630)	90 (50–130)	12 (8–20)	
DEEP (RR 1.37, 95% CrI 1.05–1.79)					
Low	250 per 1000	342 per 1000 (262–448)	92 (12–198)	11 (5–80)	Low
Moderate	350 per 1000	480 per 1000 (368–626)	130 (18–276)	8 (4–58)	
High	500 per 1000	685 per 1000 (525–895)	185 (25–395)	6 (3–40)	
Eagle eye (RR 1.23, 95% CrI 1.04–1.47)					
Low	250 per 1000	308 per 1000 (260–368)	58 (10–118)	18 (9–100)	Low
Moderate	350 per 1000	430 per 1000 (364–514)	80 (14–164)	13 (6–72)	
High	500 per 1000	615 per 1000 (520–735)	115 (20–235)	9 (4–50)	
ENDO‐AID (RR 1.26, 95% CrI 1.14–1.40)					
Low	250 per 1000	315 per 1000 (285–350)	65 (35–100)	16 (10–29)	High
Moderate	350 per 1000	441 per 1000 (399–490)	91 (49–140)	11 (7–21)	
High	500 per 1000	630 per 1000 (570–700)	130 (70–200)	8 (5–15)	
EndoAngel (RR 1.40, 95% CrI 1.11–1.77)					
Low	250 per 1000	350 per 1000 (278–442)	100 (28–192)	11 (5–37)	Low
Moderate	350 per 1000	490 per 1000 (388–619)	140 (38–269)	8 (4–26)	
High	500 per 1000	700 per 1000 (555–885)	200 (55–385)	6 (3–19)	
EndoScreener (RR 1.21, 95% CrI 1.07–1.37)					
Low	250 per 1000	302 per 1000 (268–342)	52 (18–92)	20 (11–58)	Moderate
Moderate	350 per 1000	424 per 1000 (374–480)	74 (24–130)	14 (8–41)	
High	500 per 1000	605 per 1000 (535–685)	105 (35–185)	10 (5–29)	
GI Genius (RR 1.15, 95% CrI 1.08–1.22)					
Low	250 per 1000	288 per 1000 (270–305)	37 (20–55)	27 (18–50)	High
Moderate	350 per 1000	402 per 1000 (378–427)	52 (28–77)	20 (13–36)	
High	500 per 1000	575 per 1000 (540–610)	75 (40–110)	14 (9–25)	
Henan Xuanweitang Medical (RR 1.64, 95% CrI 1.29–2.08)					
Low	250 per 1000	410 per 1000 (322–520)	160 (72–270)	7 (4–14)	Low
Moderate	350 per 1000	574 per 1000 (451–728)	224 (101–378)	5 (3–10)	
High	500 per 1000	820 per 1000 (645–1040)	320 (145–540)	4 (2–7)	
SegNet (RR 1.43, 95% CrI 1.11–1.85)					
Low	250 per 1000	358 per 1000 (278–462)	108 (28–212)	10 (5–37)	Low
Moderate	350 per 1000	500 per 1000 (388–648)	150 (38–298)	7 (3–26)	
High	500 per 1000	715 per 1000 (555–925)	215 (55–425)	5 (2–19)	

^a^ ADR with CADe = baseline ADR × risk ratio (RR).

^b^ Number needed to screen (NNS) = 1/(absolute ADR increase).

Pairwise meta‐analyses for five CADe systems were presented in Appendix S9. For CADEYE, ENDO‐AID, EndoScreener, and GI Genius, the random‐effects models demonstrated statistically significant improvements in ADR compared with control, although the magnitude of effect varied across individual studies. For EndoAngel, individual trials showed heterogeneous results with wide confidence intervals, and the pooled random‐effects estimate did not reach statistical significance, reflecting limited precision. Across all devices, the direction of effect in the pairwise analyses was broadly consistent with the results of the network meta‐analysis, supporting the robustness of the overall findings while highlighting between‐study variability within individual CADe systems.

### 3.5 Comparison of SSLDRs Across 12 CADe Devices

The network plot comparing CADe devices with controls in terms of SSLDRs is presented in Figure 2b. Figure 3b presents the forest plot comparing CADe devices with control in terms of SSLDR. Among the evaluated systems, ENDO‐AID and GI Genius suggested statistically significant improvements in SSLDR compared with control, whereas the estimates for CADEYE did not reach statistical significance.

According to the SUCRA‐based rankings (Figure 3d), ENDO‐AID ranked highest, followed by GI Genius. However, the credible intervals for these devices overlapped substantially, indicating no clear evidence of meaningful differences in SSLDR performance among them.

### 3.6 Sensitivity Analysis

To confirm the robustness of the main findings for ADR, we performed several sensitivity analyses (Appendix S10). Firstly, because withdrawal time is a well‐recognized determinant of ADR, we conducted a sensitivity analysis to minimize the potential influence of procedural time on the observed effects (Appendix S11). We excluded (1) studies with statistically significant differences in withdrawal time between groups, (2) studies that did not report withdrawal time, and (3) studies that reported withdrawal time without corresponding *p*‐values [24, 25, 30, 32, 34, 35, 36, 37, 43, 48, 51, 53, 54, 56, 60, 66, 68]. In this restricted dataset, eight of the 13 CADe devices were associated with a probable improvement in ADR compared with control (Appendix S10a).

Secondly, to evaluate whether financial or academic relationships with CADe manufacturers influenced the results, we performed a sensitivity analysis excluding studies with reported COI (Appendix S12) [23, 24, 26, 28, 29, 32, 35, 37, 38, 39, 40, 41, 43, 45, 46, 47, 49, 51, 52, 54, 57, 58, 59, 60, 62, 63, 64, 65, 67]. Within this subset, CADEYE and GI Genius were associated with estimated improvements in ADR compared with control, whereas the remaining CADe systems did not reach statistical significance (Appendix S10b).

Thirdly, to assess the robustness of the network estimates against sparse evidence, we restricted the analysis to CADe systems that had been evaluated in three or more RCTs, which resulted in the exclusion of 12 RCTs [31, 57, 58, 59, 60, 61, 62, 63, 64, 65, 66, 67]. As shown in Appendix S10c, all included CADe systems demonstrated estimated improvements in ADR compared with control, with effect estimates reaching statistical significance.

Fourthly, because CADe was evaluated under IEE conditions in only one study, we conducted a sensitivity analysis excluding this trial to examine its potential influence on the overall results. After removal of the Miyaguchi et al. study [27], the overall pattern of findings remained largely unchanged, with most CADe systems continuing to show estimated improvements in ADR compared with control (Appendix S10d).

Fifthly, given concerns that tandem‐design trials may overestimate CADe performance due to enhanced detectability during the first pass, we performed a sensitivity analysis excluding all tandem‐design studies [25, 43, 44, 46, 56, 59, 67]. As shown in Appendix S10e, restricting the analysis to parallel‐group trials did not materially alter the direction or magnitude of the estimated effects on ADR.

Sixthly, to explore the impact of study setting and generalizability, we restricted the analysis to multicenter trials, with single‐center trials excluded [21, 22, 24, 25, 26, 27, 28, 33, 34, 35, 42, 45, 46, 47, 49, 50, 51, 53, 54, 55, 58, 60, 61, 64, 65, 68]. In this sensitivity analysis, an estimated improvement in ADR reached statistical significance for GI Genius, whereas the estimates for the other CADe systems remained statistically inconclusive. Although point estimates generally favored CADe over control, the corresponding credible intervals crossed unity for most systems, indicating limited statistical certainty in the multicenter‐only setting (Appendix S10f).

Seventhly, to examine whether operator expertise modified the effects of CADe, we conducted a sensitivity analysis restricted to studies in which colonoscopies were performed exclusively by experienced endoscopists, resulting in the exclusion of RCTs that included unexperienced or mixed operators [21, 22, 24, 25, 27, 29, 30, 31, 34, 36, 37, 39, 41, 43, 44, 45, 50, 51, 55, 56, 59, 61, 65, 66, 68]. In this analysis, several CADe systems were associated with statistically significant estimated improvements in ADR (Appendix S10g).

Finally, to explore potential generation effects of CADe systems, we restricted the analysis to studies published from 2023 onward, using publication year as a proxy for more recent CADe technologies, which resulted in the exclusion of earlier RCTs [29, 39, 40, 43, 44, 45, 46, 47, 54, 55, 59, 61, 63, 64, 65]. In this analysis, several CADe systems were associated with statistically significant estimated improvements in ADR compared with control, and the overall direction and magnitude of the effect estimates were consistent with those observed in the primary analysis (Appendix S10h).

Taken together, across the eight sensitivity analyses, GI Genius tended to show estimated improvements in ADR that reached statistical significance in all analyses, whereas CADEYE suggested statistically significant estimated improvements in seven of the eight analyses, and ENDO‐AID also suggested significant estimates in six.

## 4 Discussion

This systematic review and network meta‐analysis provides an integrated comparison of currently available CADe systems in colonoscopy, addressing a clinically relevant question that could not be resolved by conventional pairwise meta‐analyses. By synthesizing 48 RCT and nearly 39,000 patients, our study offers a comprehensive overview of the relative performance of multiple AI‐based platforms across diverse settings. Overall, several CADe systems demonstrated meaningful improvements in ADR compared with standard colonoscopy, while the overlapping CrIs across devices suggest no clear evidence of superiority among them. When translated into NNS, these absolute gains corresponded to approximately one additional adenoma detected per 10–30 colonoscopies across low‐ to high‐risk baseline scenarios, highlighting the practical clinical relevance of CADe‐assisted colonoscopy. Improvements were also observed for SSL detection in some CADe devices. Together, these findings support the concept that CADe‐assisted colonoscopy may enhance detection performance beyond conventional practice, while highlighting that currently available systems appear broadly comparable in efficacy.

For adenoma detection, the CINeMA assessment indicated high confidence in the estimates for CADEYE, GI Genius, and ENDO‐AID. These three platforms were supported by the largest bodies of randomized evidence, demonstrated narrower CrIs, and showed no major concerns regarding within‐study bias, indirectness, or heterogeneity. This combination of abundant data and methodological consistency allowed their estimated effects to remain stable across analytical assumptions. To complement relative comparisons, our risk‐difference network meta‐analysis showed that CADe implementation increased ADR by approximately 5–10 percentage points, depending on the system evaluated. Presenting effects in absolute terms facilitates clinical interpretation, underscoring that even modest relative gains may translate into clinically meaningful increases in the detection of precancerous lesions.

The robustness of these findings was further supported by eight sensitivity analyses addressing procedural factors, study design, operator expertise, study setting, potential conflicts of interest, and the timing of technological implementation. Across these complementary analyses, the overall direction and magnitude of effect estimates remained broadly consistent with the primary network meta‐analysis, indicating that improvements in ADR with CADe were not driven by isolated subgroups or methodological artifacts. Although statistical significance fluctuated when analyses were restricted to narrower evidence bases—such as multicenter trials or experienced operators only—the trend consistently favored CADe over standard colonoscopy. Collectively, these results reinforce that the observed benefits are durable across heterogeneous clinical environments and analytic assumptions, lending additional credibility to the main conclusion that CADe meaningfully enhances adenoma detection.

Regarding SSLDRs, GI Genius and ENDO‐AID probably increase detection compared to the control. Generally, the detection of SSLs is more challenging than that of adenomas because of their subtle color contrast and their frequent location in the ascending colon, which has multiple deep haustra. Despite the subtle characteristics of SSLs, the risk of colorectal cancer associated with SSLs is comparable to that of adenomas [70]. Separate investigations of ADRs and SSLDRs are therefore necessary to clarify the usefulness of CADe.

We acknowledged some limitations of this systematic review. First, version changes in CADe systems were not considered. For example, Troya et al. reported progressive improvements in the sensitivity of GI Genius [71]. Moreover, the operational mode of CADe devices may also influence ADRs. Given the limited reporting of software versions across trials, publication year was pragmatically used as a surrogate marker of CADe generation, acknowledging that this approach cannot fully capture within‐platform technical evolution. Second, important factors such as country, experience of endoscopists, quality of bowel preparation, and withdrawal time were not unified across studies. Third, the cost of CADe systems was not assessed; therefore, a cost‐effective analysis could not be performed. Fourth, direct head‐to‐head comparisons across CADe devices were not assessed. Fifth, stratified analyses by screening indication could not be performed because this information was inconsistently and insufficiently reported across the included trials, limiting assessment of indication‐specific effect modification. Sixth, although the control intervention was generally defined as standard white‐light colonoscopy without CADe, minor variations in procedural protocols across trials cannot be fully excluded. In addition, one included study [27] evaluated CADe in combination with linked color imaging, which may limit the uniformity of indirect comparisons.

In conclusion, several CADe systems were associated with improved ADR compared with standard colonoscopy. Based on the CINeMA assessment, moderate confidence evidence supports the effectiveness of specific CADe technologies in enhancing ADR. These findings suggest that selected CADe systems may provide clinically meaningful benefits in routine colonoscopy practice, while the magnitude of benefit may vary across platforms and clinical settings. Head‐to‐head RCTs are still required to directly compare the effects among different CADe devices.

## Author Contributions

**Satoshi Shinozaki:** conception and design, data collection, data analysis and interpretation, methodology, investigation, writing – original draft, writing – review and editing, and final approval of the manuscript. **Jun Watanabe:** conception and design, data collection, data analysis and interpretation, methodology, investigation, writing – original draft, writing – review and editing, revised it critically for important intellectual content, and final approval of the manuscript. **Takeshi Kanno:** data analysis and interpretation, methodology, writing – review and editing, revised it critically for important intellectual content, and final approval of the manuscript. **Katsuyuki Nakazawa:** data analysis and interpretation, writing – review and editing, revised it critically for important intellectual content, and final approval of the manuscript. **Tomonori Yano:** supervision, writing – review and editing, revised it critically for important intellectual content, and final approval of the manuscript.

## Funding

The authors have nothing to report.

## Conflicts of Interest

Tomonori Yano is affiliated with a department endowed by Fujifilm Corporation and has received lecture honoraria from Fujifilm Corporation. The sponsor had no role in the design, conduct, analysis, or interpretation of this study. All other authors declare no relevant conflicts of interest.

## Supporting information


**Appendix S1:** PRISMA checklist.
**Appendix S2:** Search strategy.
**Appendix S3:** Assessment of transitivity: distribution of effect modifiers.
**Appendix S4:** Indications of colonoscopies.
**Appendix S5:** Summary of confidence in network estimates in ADRs.
**Appendix S6:** Assessment of potential publication bias and small‐study effects.
**Appendix S7:** SUCRA‐based ranking.
**Appendix S8:** Risk difference‐based network meta‐analysis of ADR.
**Appendix S9:** Pairwise meta‐analyses of adenoma detection rate (ADR) for individual CADe systems.
**Appendix S10:** Forest plots showing the results of sensitivity analyses evaluating the robustness of the primary network meta‐analysis.
**Appendix S11:** Withdrawal time in the included studies.
**Appendix S12:** Disclosure of conflicts of interest (COI) related to manufacturers of CADe devices used in the included studies.

## Data Availability

Study materials will be shared upon reasonable request, after consultation and agreement of the authors.
