# Supplementary material for: Comparative Performance of Artificial Intelligence‐Based Computer‐Aided Detection Systems for Colorectal Polyps: A Systematic Review and Network Meta‐Analysis
Source: Dig Endosc. 2026 Mar 29;38(4):e70138. doi: 10.1111/den.70138 (PMC13033948; doi:10.1111/den.70138)
Supplement: Supplementary file 1 — Appendix S1: PRISMA checklist. Appendix S2: Search strategy. Appendix S3: Assessment of transitivity: distribution of effect modifiers. Appendix S4: Indications of colonoscopies. Appendix S5: Summary of confidence in network estimates in ADRs. Appendix S6: Assessment of potential publication bias and small‐study effects. Appendix S7: SUCRA‐based ranking. Appendix S8: Risk difference‐based network meta‐analysis of ADR. Appendix S9: Pairwise meta‐analyses of adenoma detection rate (ADR) for individual CADe systems. Appendix S10: Forest plots showing the results of sensitivity analyses evaluating the robustness of the primary network meta‐analysis. Appendix S11: Withdrawal time in the included studies. Appendix S12: Disclosure of conflicts of interest (COI) related to manufacturers of CADe devices used in the included studies. [file DEN-38-0-s001.docx]

**Supporting information**

**Appendix S1:** Checklist of Items to Include When Reporting a Systematic Review Involving a Network Meta-analysis

| **Section/Topic** | **Item #** | **Checklist Item** | **Reported on Page #** |
| --- | --- | --- | --- |
| **TITLE** |  |  |  |
| Title | 1 | Identify the report as a systematic review *incorporating a network meta-analysis (or related form of meta-analysis).* | 1 |
|  |  |  |  |
| **ABSTRACT** |  |  |  |
| Structured summary | 2 | Provide a structured summary including, as applicable:  **Background:** main objectives  **Methods:** data sources; study eligibility criteria, participants, and interventions; study appraisal; and *synthesis methods, such as network meta-analysis.*  **Results:** number of studies and participants identified; summary estimates with corresponding confidence/credible intervals; *treatment rankings may also be discussed. Authors may choose to summarize pairwise comparisons against a chosen treatment included in their analyses for brevity.*  **Discussion/Conclusions:** limitations; conclusions and implications of findings.  **Other:** primary source of funding; systematic review registration number with registry name. | 4 |
|  |  |  |  |
| **INTRODUCTION** |  |  |  |
| Rationale | 3 | Describe the rationale for the review in the context of what is already known*, including mention of why a network meta-analysis has been conducted.* | 6 |
| Objectives | 4 | Provide an explicit statement of questions being addressed, with reference to participants, interventions, comparisons, outcomes, and study design (PICOS). | 7 |
|  |  |  |  |
| **METHODS** |  |  |  |
| Protocol and registration | 5 | Indicate whether a review protocol exists and if and where it can be accessed (e.g., Web address); and, if available, provide registration information, including registration number. | 8 |
| Eligibility criteria | 6 | Specify study characteristics (e.g., PICOS, length of follow-up) and report characteristics (e.g., years considered, language, publication status) used as criteria for eligibility, giving rationale. *Clearly describe eligible treatments included in the treatment network, and note whether any have been clustered or merged into the same node (with justification).* | 8 |
| Information sources | 7 | Describe all information sources (e.g., databases with dates of coverage, contact with study authors to identify additional studies) in the search and date last searched. | 9 |
| Search | 8 | Present full electronic search strategy for at least one database, including any limits used, such that it could be repeated. | 9 |
| Study selection | 9 | State the process for selecting studies (i.e., screening, eligibility, included in systematic review, and, if applicable, included in the meta-analysis). | 11 |
| Data collection process | 10 | Describe method of data extraction from reports (e.g., piloted forms, independently, in duplicate) and any processes for obtaining and confirming data from investigators. | 11 |
| Data items | 11 | List and define all variables for which data were sought (e.g., PICOS, funding sources) and any assumptions and simplifications made. | 12 |
| **Geometry of the network** | **S1** | Describe methods used to explore the geometry of the treatment network under study and potential biases related to it. This should include how the evidence base has been graphically summarized for presentation, and what characteristics were compiled and used to describe the evidence base to readers. | NA |
| Risk of bias within individual studies | 12 | Describe methods used for assessing risk of bias of individual studies (including specification of whether this was done at the study or outcome level), and how this information is to be used in any data synthesis. | 12 |
| Summary measures | 13 | State the principal summary measures (e.g., risk ratio, difference in means). *Also describe the use of additional summary measures assessed, such as treatment rankings and surface under the cumulative ranking curve (SUCRA) values, as well as modified approaches used to present summary findings from meta-analyses.* | 13 |
| Planned methods of analysis | 14 | Describe the methods of handling data and combining results of studies for each network meta-analysis. This should include, but not be limited to:   - *Handling of multi-arm trials;* - *Selection of variance structure;* - *Selection of prior distributions in Bayesian analyses; and* - *Assessment of model fit.* | 12 |
| **Assessment of Inconsistency** | **S2** | Describe the statistical methods used to evaluate the agreement of direct and indirect evidence in the treatment network(s) studied. Describe efforts taken to address its presence when found. | 12 |
| Risk of bias across studies | 15 | Specify any assessment of risk of bias that may affect the cumulative evidence (e.g., publication bias, selective reporting within studies). | 13 |
| Additional analyses | 16 | Describe methods of additional analyses if done, indicating which were pre-specified. This may include, but not be limited to, the following:   - Sensitivity or subgroup analyses; - Meta-regression analyses; - *Alternative formulations of the treatment network; and* - *Use of alternative prior distributions for Bayesian analyses (if applicable).* | 15 |
|  |  |  |  |
| **RESULTS†** |  |  |  |
| Study selection | 17 | Give numbers of studies screened, assessed for eligibility, and included in the review, with reasons for exclusions at each stage, ideally with a flow diagram. | 17 |
| **Presentation of network structure** | **S3** | Provide a network graph of the included studies to enable visualization of the geometry of the treatment network. | 20 |
| **Summary of network geometry** | **S4** | Provide a brief overview of characteristics of the treatment network. This may include commentary on the abundance of trials and randomized patients for the different interventions and pairwise comparisons in the network, gaps of evidence in the treatment network, and potential biases reflected by the network structure. | 20 |
| Study characteristics | 18 | For each study, present characteristics for which data were extracted (e.g., study size, PICOS, follow-up period) and provide the citations. | 17 |
| Risk of bias within studies | 19 | Present data on risk of bias of each study and, if available, any outcome level assessment. | 18 |
| Results of individual studies | 20 | For all outcomes considered (benefits or harms), present, for each study: 1) simple summary data for each intervention group, and 2) effect estimates and confidence intervals. *Modified approaches may be needed to deal with information from larger networks.* | 19 |
| Synthesis of results | 21 | Present results of each meta-analysis done, including confidence/credible intervals. *In larger networks, authors may focus on comparisons versus a particular comparator (e.g. placebo or standard care), with full findings presented in an appendix. League tables and forest plots may be considered to summarize pairwise comparisons.* If additional summary measures were explored (such as treatment rankings), these should also be presented. | 20 |
| **Exploration for inconsistency** | **S5** | Describe results from investigations of inconsistency. This may include such information as measures of model fit to compare consistency and inconsistency models, *P* values from statistical tests, or summary of inconsistency estimates from different parts of the treatment network. | NA |
| Risk of bias across studies | 22 | Present results of any assessment of risk of bias across studies for the evidence base being studied. | 15 |
| Results of additional analyses | 23 | Give results of additional analyses, if done (e.g., sensitivity or subgroup analyses, meta-regression analyses*, alternative network geometries studied, alternative choice of prior distributions for Bayesian analyses,* and so forth). | 22 |
|  |  |  |  |
| **DISCUSSION** |  |  |  |
| Summary of evidence | 24 | Summarize the main findings, including the strength of evidence for each main outcome; consider their relevance to key groups (e.g., healthcare providers, users, and policy-makers). | 25 |
| Limitations | 25 | Discuss limitations at study and outcome level (e.g., risk of bias), and at review level (e.g., incomplete retrieval of identified research, reporting bias). *Comment on the validity of the assumptions, such as transitivity and consistency. Comment on any concerns regarding network geometry (e.g., avoidance of certain comparisons).* | 29 |
| Conclusions | 26 | Provide a general interpretation of the results in the context of other evidence, and implications for future research. | 30 |
|  |  |  |  |
| **FUNDING** |  |  |  |
| Funding | 27 | Describe sources of funding for the systematic review and other support (e.g., supply of data); role of funders for the systematic review. This should also include information regarding whether funding has been received from manufacturers of treatments in the network and/or whether some of the authors are content experts with professional conflicts of interest that could affect use of treatments in the network. | 3 |

**Appendix S2: Search strategy**

We conducted a comprehensive bibliographic search of the following databases from 2015 to August 16, 2025: MEDLINE (Ovid), Embase (Ovid), and the Cochrane Central Register of Controlled Trials (CENTRAL) (Ovid). We searched for text words as well as controlled vocabulary (MeSH or Emtree terms) related to colonic polyps or adenomas, colonoscopy or endoscopy, and computer-aided detection (CADe) systems, using Boolean operators (AND, OR, NOT). The search strategy was first developed for MEDLINE and then translated to Embase and CENTRAL.

<MEDLINE via PubMed search strategy>

#1. ("colonoscopy"[MeSH Terms] OR colonoscop*[tiab] OR coloscop*[tiab] OR ileocolonoscop*[tiab] OR sigmoidoscop*[tiab] OR proctosigmoidoscop*[tiab] OR ((colon*[tiab] OR rectum[tiab] OR rectal[tiab] OR colorectal[tiab] OR colo-rectal[tiab] OR intestin*[tiab] OR bowel[tiab]) AND endoscop*[tiab]))

#2. ("computer-aided detection"[tiab] OR "computer-assisted detection"[tiab] OR CADe[tiab] OR "real-time detection"[tiab] OR "detection system"[tiab] OR "detection systems"[tiab])

#3. ("2015/01/01"[Date - Publication] : "2025/09/03"[Date - Publication])

#4. #1 AND #2 AND #3

<EMBASE search strategy>

S1. (TI(colonoscop*) OR AB(colonoscop*) OR TI(coloscop*) OR AB(coloscop*) OR TI(ileocolonoscop*) OR AB(ileocolonoscop*) OR TI(sigmoidoscop*) OR AB(sigmoidoscop*) OR TI(proctosigmoidoscop*) OR AB(proctosigmoidoscop*) OR ((TI(colon*) OR AB(colon*) OR TI(rectum) OR AB(rectum) OR TI(rectal) OR AB(rectal) OR TI(colorectal) OR AB(colorectal) OR TI(colo-rectal) OR AB(colo-rectal) OR TI(intestin*) OR AB(intestin*) OR TI(bowel) OR AB(bowel)) AND (TI(endoscop*) OR AB(endoscop*))) OR EMB.EXACT.EXPLODE("colonoscopy"))

S2. (TI("computer-aided detection") OR AB("computer-aided detection") OR TI("computer-assisted detection") OR AB("computer-assisted detection") OR TI(CADe) OR AB(CADe) OR TI("real-time detection") OR AB("real-time detection") OR TI("detection system") OR AB("detection system") OR TI("detection systems") OR AB("detection systems"))

S3. (PY >= 2015 AND PY <= 2025)

S4. S1 AND S2 AND S3

<CENTRAL search strategy>

1. (([mh colonoscopy] OR colonoscop*:ti,ab OR coloscop*:ti,ab OR ileocolonoscop*:ti,ab OR sigmoidoscop*:ti,ab OR proctosigmoidoscop*:ti,ab OR ((colon*:ti,ab OR rectum:ti,ab OR rectal:ti,ab OR colorectal:ti,ab OR colo-rectal:ti,ab OR intestin*:ti,ab OR bowel:ti,ab) AND endoscop*:ti,ab))) AND (("computer-aided detection":ti,ab OR "computer-assisted detection":ti,ab OR CADe:ti,ab OR "real-time detection":ti,ab OR "detection system":ti,ab OR "detection systems":ti,ab))

**Appendix S3**: Assessment of transitivity: distribution of effect modifiers

To assess the plausibility of the transitivity assumption in this network meta-analysis comparing CADe devices with control, we evaluated whether the included trials were sufficiently similar in terms of key characteristics that could potentially modify the treatment effect. Specifically, we assessed the distribution of the following effect modifiers: country, age, and the number of experienced and inexperienced endoscopists per study arm. These variables were selected based on their potential influence on adenoma detection and sessile serrated polyp detection rates. The distribution of these effect modifiers across different intervention groups was examined visually using the tabulated data below. The overall similarity in these characteristics across trials supports the assumption of transitivity required for valid indirect comparisons.

| **First author** | **CADe device** | **Country** | **Mean age, year (CADe/control)** | **Number of experienced endoscopists** | **Number of inexperienced endoscopists** |
| --- | --- | --- | --- | --- | --- |
| Alali | CADEYE | Kuwait | 51/54 | NR | NR |
| Aniwan | CADEYE | Thailand | 63/62 | 7 | 10 |
| Desai | CADEYE | US | 59/59 | 30 | 0 |
| Djinbachian | CADEYE | Canada | 64/64 | 4 | 1 |
| Hiratsuka | CADEYE | Japan | 67/71 | NR | NR |
| Hüneburg | CADEYE | Germany | 50/46 | 3 | 0 |
| Miyaguchi | CADEYE | Japan | 65/66 | 3 | 2 |
| Nakashima | CADEYE | Japan | 55/56 | 3 | 0 |
| Rondonotti | CADEYE | Italy | 62/61* | NR | NR |
| Tiankanon | CADEYE | Thailand | 63/62 | 18 | 13 |
| Yamaguchi | CADEYE | Japan | 63/63 | 0 | 6 |
| Zimmermann-Fraedrich | CADEYE | Germany | 63/63 | 43 | 0 |
| Ahmad | GI Genius | German | 66/66 | 8 | 0 |
| Engelke | GI Genius | German | 66/67* | NR | NR |
| Karsenti | GI Genius | France | 58/58 | 20 | 0 |
| Lagström | GI Genius | Denmark | 64/63 | NR | NR |
| Mangas-Sanjuan | GI Genius | Spain | 61/61 | NR | NR |
| Ortiz | GI Genius | Spain, Germany, Italy, Belgium | 48/50 | 30 | 0 |
| Repici, Gut | GI Genius | Italy | 62/63 | 0 | 10 |
| Repici, Gastro | GI Genius | Italy | 62/61 | 6 | 0 |
| Seager | GI Genius | UK | 63/62 | NR | NR |
| Thiruvengadam | GI Genius | US | 56/64* | 6 | 0 |
| Wallace | GI Genius | UK, US, Italy | 63/65 | NR | NR |
| Glissen Brown | EndoScreener | US | 61/61 | NR | NR |
| Liu, Ther Adv | EndoScreener | China | 50/49 | 8 | 3 |
| Wang, Gastro | EndoScreener | China | 47/48 | 3 | 0 |
| Wang, Lancet | EndoScreener | China | 49/49 | 4 | 0 |
| Wang, GR | EndoScreener | China | 46/47* | 8 | 0 |
| Gimeno-García | ENDO-AID | Spain | 63/65 | 8 | 0 |
| Lui | ENDO-AID | Hong Kong | 66/66 | 6 | 6 |
| Lau | ENDO-AID | Hong Kong | 66/65 | 0 | 22 |
| Spada | ENDO-AID | Italy | 62/62 | 14 | 0 |
| Vilkoite | ENDO-AID | Latvia | 50/51 | 2 | 0 |
| Gong | EndoAngel | China | 50/49 | 6 | 0 |
| Yao, Endosc | EndoAngel | China | 51/51 | NR | NR |
| Yao, GIE | EndoAngel | China | 51/50 | 10 | 8 |
| Wei, AJG | EndoVigilant | US | 58/58 | 7 | 0 |
| Wei, AIMI | EndoVigilant | US | 69/68 | 4 | 0 |
| Kamba | modified YOLOv3 (LPIXEL) | Japan | 62/61 | 22 | 10 |
| Lachter | DEEP | Israel | 61/61 | 7 | 0 |
| Liu, Saudi | Henan Xuanweitang Medical | China | 51/50 | NR | NR |
| Maas, Endosc | DISCOVERY | Europe, Canada | 61/61* | 14 | 0 |
| Shaukat | SKOUT | US | 61/60 | 22 | 0 |
| Su | AQCS | China | 51/52 | 6 | 0 |
| Wang, Gut | SegNet | China | 51/50 | 4 | 4 |
| Xu | Eagle-Eye | China | 57/57 | 12 | 12 |
| Maas, Lancet | MAGENTIQ-COLO | Europe, US, Israel | 60/60 | 31 | 0 |
| Yabuuchi | EndoBRAIN-EYE | Japan | 69/68 | 8 | 10 |

GR: Gastroenterology Reports, GIE: Gastrointestinal Endoscopy, NR: not reported

* Median age

Appendix S4. Indications of colonoscopies

| First author | Screening | Surveillance | Symptomatic | FIT positive |
| --- | --- | --- | --- | --- |
| Alali | 94% | 6% | 0% | 0% |
| Aniwan | 88-89% | 0% | 0% | 11-12% |
| Desai | 50-55% | 45-50% | 0% | 0% |
| Djinbachian | 14-16% | 53-55% | 18-22% | 7% |
| Hiratsuka | 59-60% | 24-31% | 8-17% | 0% |
| Hüneburg | 0% | 100% | 0% | 0% |
| Miyaguchi | 27-28% | 19% | 21% | 32-34% |
| Nakashima | 7-8% | 36-40% | 0% | 52-57% |
| Rondonotti | 0% | 0% | 0% | 100% |
| Tiankanon | 100% | 0% | 0% | 0% |
| Yamaguchi | 29-26% | 11-13% | 9-12% | 47-54% |
| Zimmermann-Fraedrich | 72% | NR | NR | NR |
| Ahmad | 0% | 0% | 0% | 100% |
| Engelke | 4% | 8% | 88% | 0% |
| Karsenti | 14-16% | 25-28% | 25-27% | 6-8% |
| Lagström | 28-29% | 14-22% | 0% | 40-41% |
| Mangas-Sanjuan | 0% | 0% | 0% | 100% |
| Ortiz | 0% | 100%* | 0 | 0 |
| Repici, Gut | 29-30% | 36-39% | 25-29% | 7% |
| Repici, Gastro | 22-23% | 23-25% | 22-25% | 30-31% |
| Seager | 61% | 0% | 39% | 0% |
| Thiruvengadam | 65-69% | 6-10% | 6% | 19% |
| Wallace | 35-36% | 64-65% | 0% | 0% |
| Glissen Brown | 60% | 40% | 0% | 0% |
| Liu, Ther Adv | 21-25% | 0% | 75-79% | 0% |
| Wang, Gastro | 36-37% | 63-64% | 0% | 0% |
| Wang, Lancet | 16-17% | 0% | 83-84% | 0% |
| Wang, GR | 16-18% | 5-6% | 77－78% | 0% |
| Gimeno-García | 32-36% | 32-33% | 35% | 0% |
| Lui | 18% | 28% | 54% | 0% |
| Lau | 6-7% | 32-33% | 60-62% | 0% |
| Spada | 55-59% | 18-20% | 22-25% | 0% |
| Vilkoite | NR | NR | NR | NR |
| Gong | 17-18% | 4-6% | 76-79% | 0% |
| Yao, Endosc | 89% | 10% | 1% | 0% |
| Yao, GIE | 64% | 7-10% | 26-30% | 0% |
| Wei, AJG | 72% | 28% | 0% | 0% |
| Wei, AIMI | 11% | 89% | 0% | 0% |
| Kamba | 0% | 33-38% | 44-49% | 17-18% |
| Lachter | 32% | 35% | 31% | NR |
| Liu, Saudi | 7% | 0% | 93% | 0% |
| Maas, Endosc | 19-20% | 39-42% | 39-41% | 0% |
| Shaukat | 64-67% | 33-36% | 0% | 0% |
| Su | NR | NR | 63-68% | 0% |
| Wang, Gut | 8% | 0% | 92% | 0% |
| Xu | NR | NR | NR | NR |
| Maas, Lancet | 56% | 44% | 0% | 0% |
| Yabuuchi | 28% | 42-43% | 17-18% | 12% |

FIT: fecal immunochemical test, NR: not reported

* Patients with Lynch syndrome

Appendix S5. Summary of confidence in network estimates in ADRs.

| Device | Study, n | Within-study bias | Reporting bias | Indirectness | Imprecision | Heterogeneity | Incoherence | Confidence rating |
| --- | --- | --- | --- | --- | --- | --- | --- | --- |
| AQCS | 1 | Some concerns | Some concerns | No concerns | Major concerns | No concerns | Not applicable | Low |
| CADEYE | 12 | No concerns | Low risk | No concerns | No concerns | No concerns | Not applicable | High |
| DEEP | 1 | Some concerns | Some concerns | No concerns | Major concerns | No concerns | Not applicable | Low |
| DISCOVERY | 1 | Some concerns | Some concerns | No concerns | Major concerns | No concerns | Not applicable | Low |
| ENDO-AID | 5 | No concerns | Low risk | No concerns | No concerns | No concerns | Not applicable | High |
| Eagle-Eye | 1 | Some concerns | Some concerns | No concerns | Major concerns | Some concerns | Not applicable | Low |
| EndoAngel | 3 | Some concerns | Some concerns | No concerns | Major concerns | No concerns | Not applicable | Low |
| EndoBRAIN-EYE | 1 | Some concerns | Some concerns | No concerns | Major concerns | No concerns | Not applicable | Low |
| EndoScreener | 5 | Some concerns | Low risk | No concerns | Some concerns | No concerns | Not applicable | Moderate |
| EndoVigilant | 2 | Some concerns | Some concerns | No concerns | Major concerns | No concerns | Not applicable | Low |
| GI Genius | 10 | No concerns | Low risk | No concerns | No concerns | Some concerns | Not applicable | High |
| Henan Xuanweitang Medical | 1 | Some concerns | Some concerns | No concerns | Major concerns | No concerns | Not applicable | Low |
| MAGENTIQ-COLO | 1 | Some concerns | Some concerns | No concerns | Major concerns | Some concerns | Not applicable | Low |
| SKOUT | 1 | Some concerns | Some concerns | No concerns | Major concerns | No concerns | Not applicable | Low |
| SegNet | 1 | Some concerns | Some concerns | No concerns | Major concerns | No concerns | Not applicable | Low |
| modified YOLOv3 (LPIXEL) | 1 | Some concerns | Some concerns | No concerns | Major concerns | No concerns | Not applicable | Low |

Appendix S6. Assessment of potential publication bias and small-study effects.

1. Comparison-adjusted funnel plot


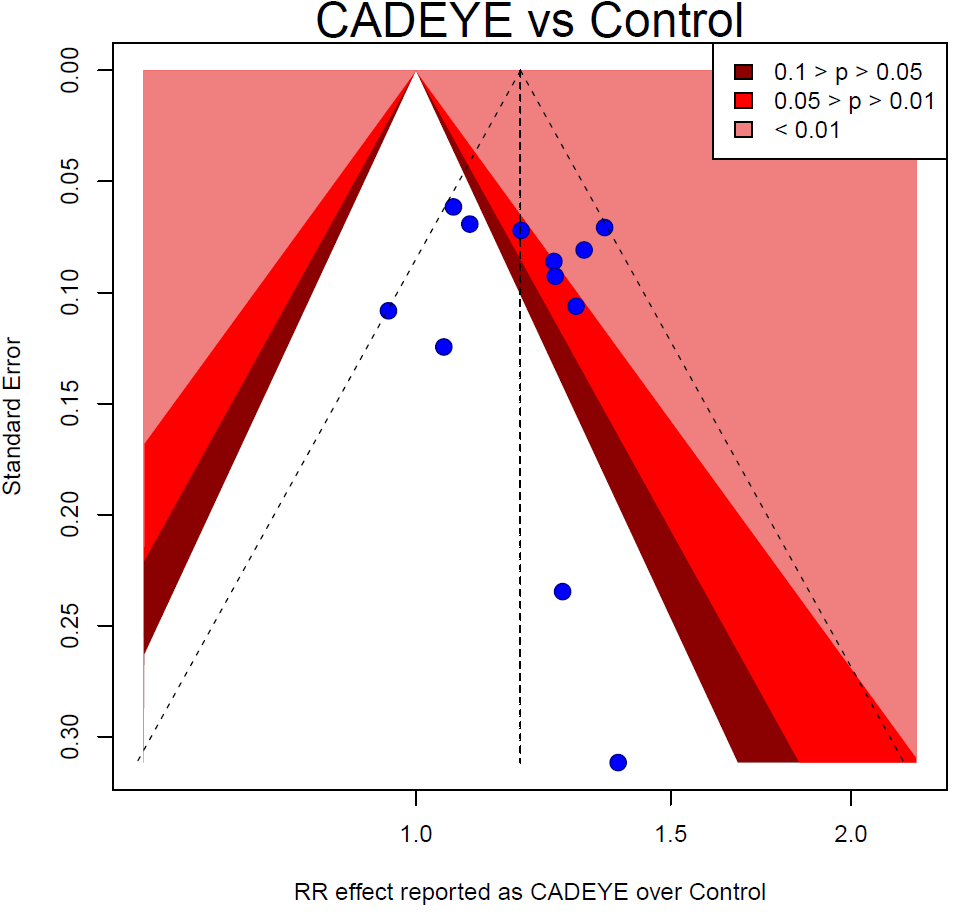


Egger test: p = 0.78


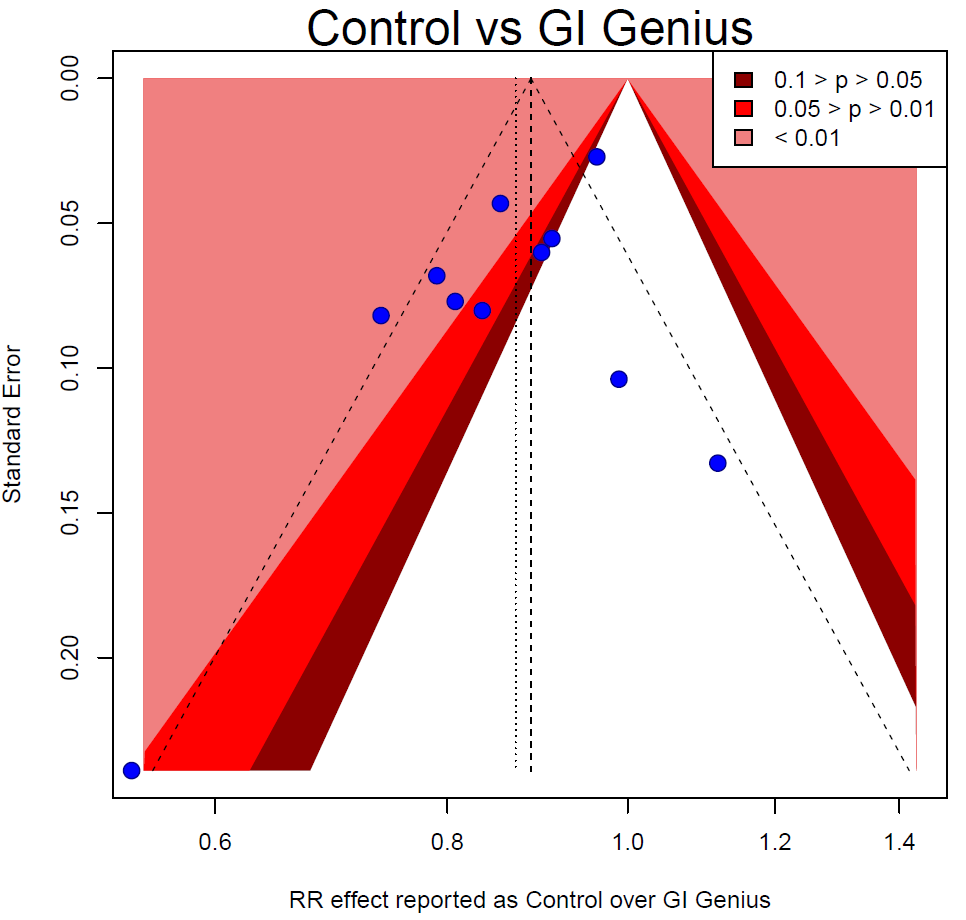


Egger test: p = 0.11

1. Network meta-regression for variance of the (linear) treatment effect


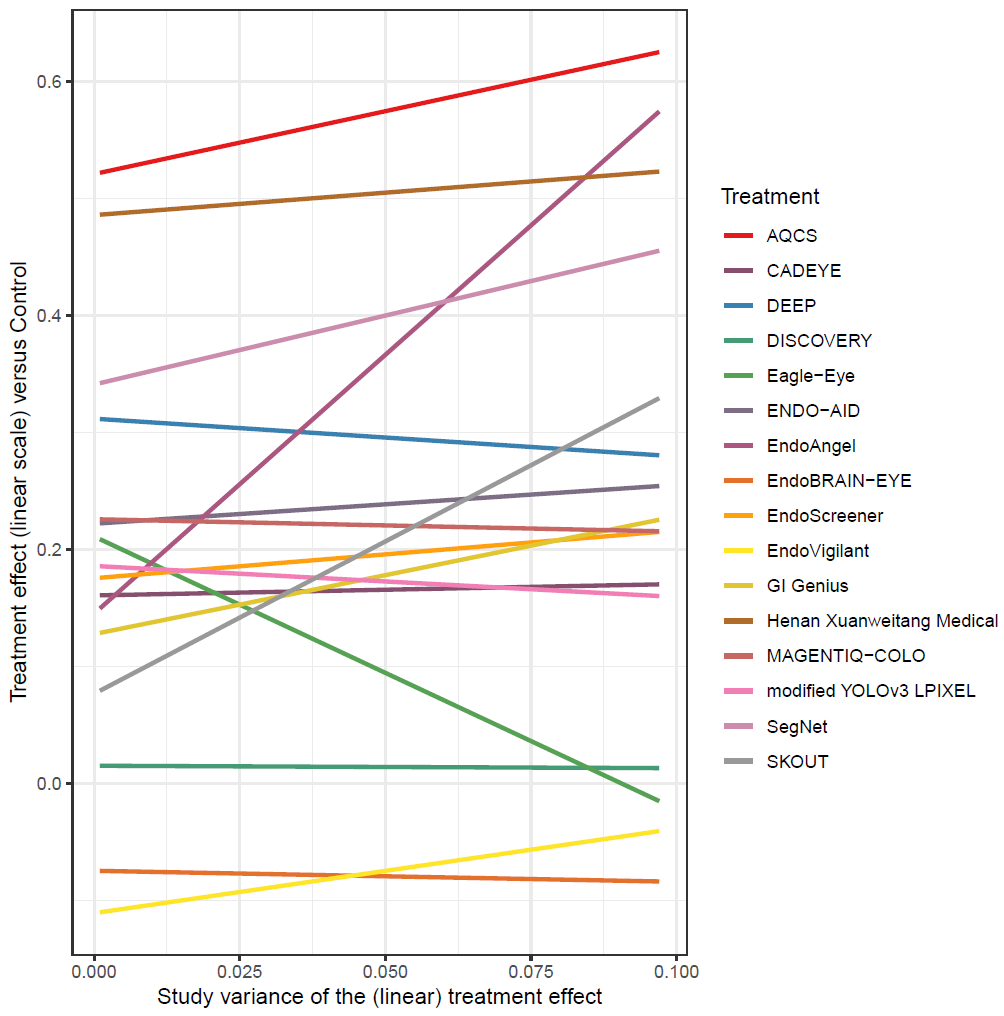


Appendix S7. SUCRA-based ranking

| System | SUCRA |
| --- | --- |
| AQCS | 93.8 |
| CADEYE | 45.4 |
| Control | 15.1 |
| DEEP | 71.7 |
| DISCOVERY | 21.8 |
| Eagle Eye | 55.1 |
| ENDO-AID | 61.0 |
| EndoAngel | 75.3 |
| EndoBRAIN-EYE | 8.3 |
| EndoScreener | 51.2 |
| EndoVigilant | 5.2 |
| GI Genius | 37.6 |
| Henan Xuanweitang Medical | 91.3 |
| MAGENTIQ COLO | 57.7 |
| Modified YOLOv3 LPIXEL | 50.7 |
| SegNet | 77.8 |
| SKOUT | 30.1 |

Appendix S8. Risk difference–based network meta-analysis of ADR. Forest plot showing absolute differences in ADR between each CADe device and control, estimated using a frequentist random-effects model. Squares represent point estimates of risk difference (RD), with horizontal lines indicating 95% confidence intervals (CIs). Positive values indicate higher ADR compared with control.


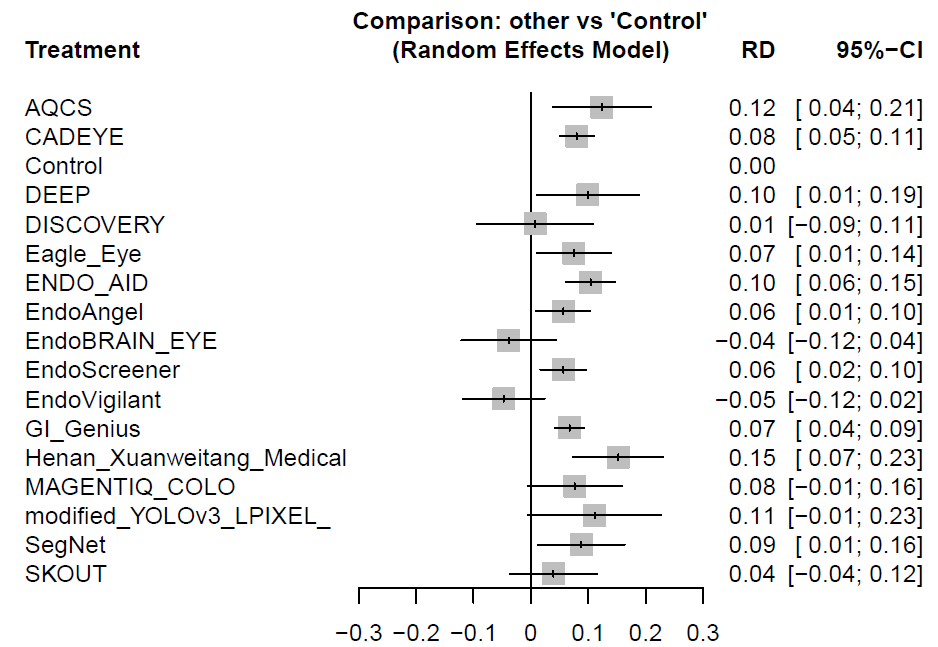


Appendix S9. Pairwise meta-analyses of adenoma detection rate (ADR) for individual CADe systems with three or more RCTs. Forest plots of randomized controlled trials comparing each CADe system with control in terms of ADR. Individual study estimates and pooled estimates from random-effects (primary) and fixed-effect models are shown. Risk ratios (RRs) are presented on a logarithmic scale, with horizontal lines indicating 95% confidence intervals. The vertical dashed line indicates no difference (RR = 1.0).

(a) CADEYE


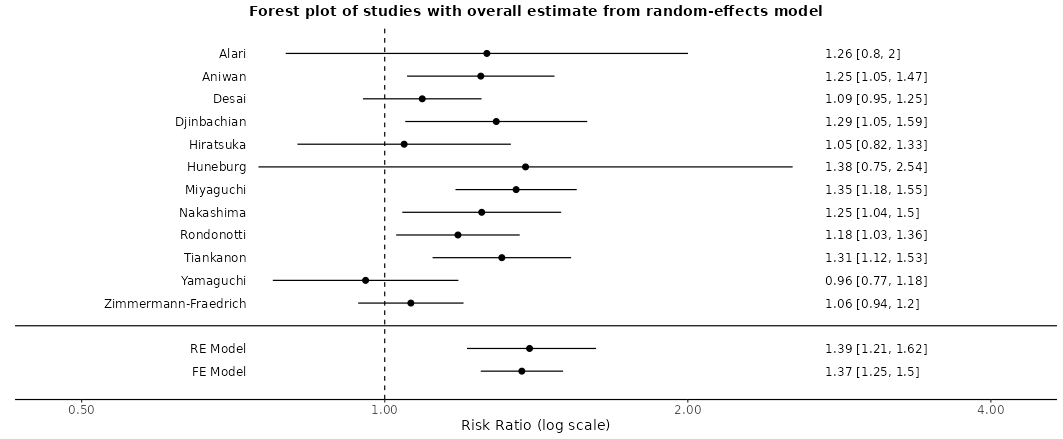


(b) EndoAngel


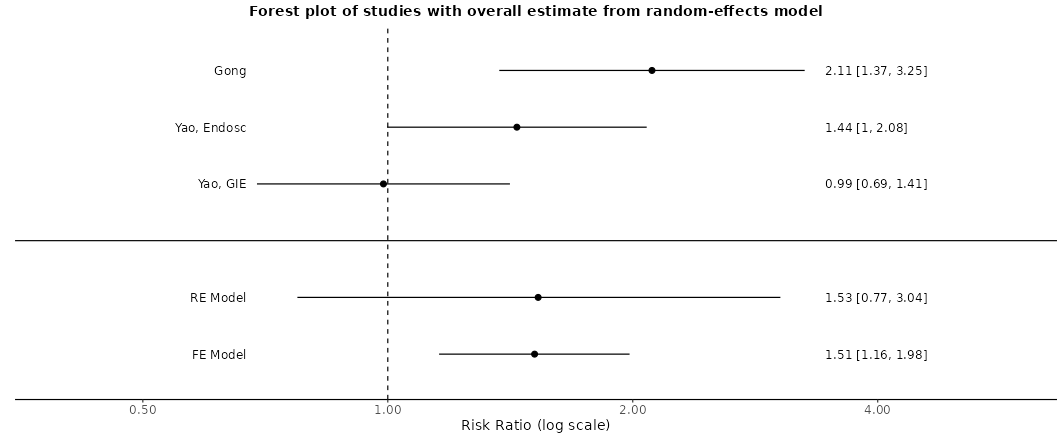


(c) ENDO-AID


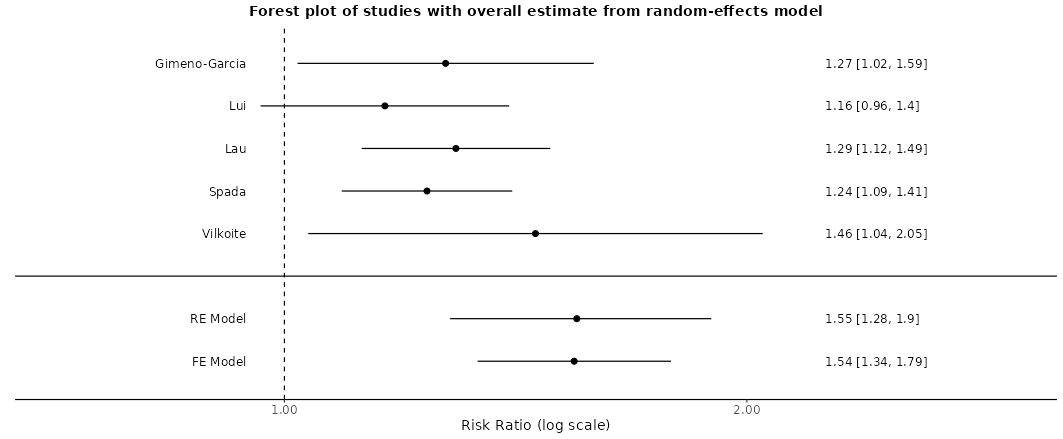


(d) EndoScreener


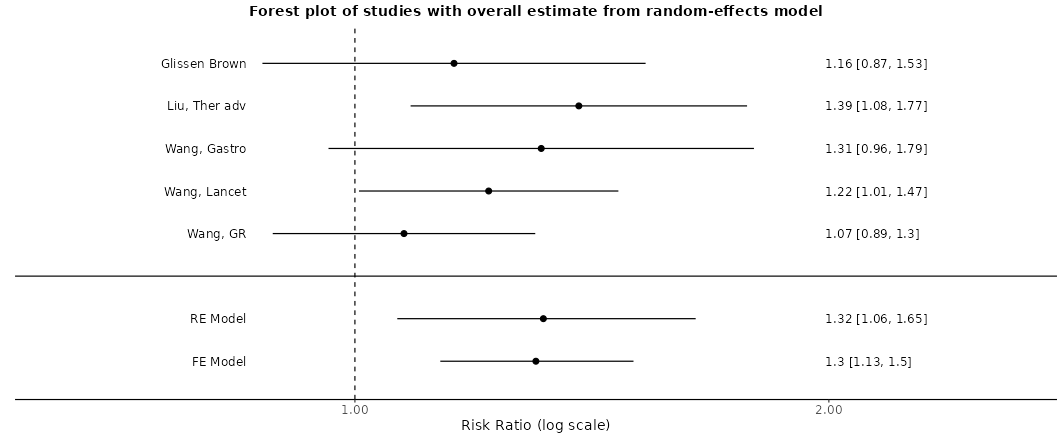


(e) GI Genius.


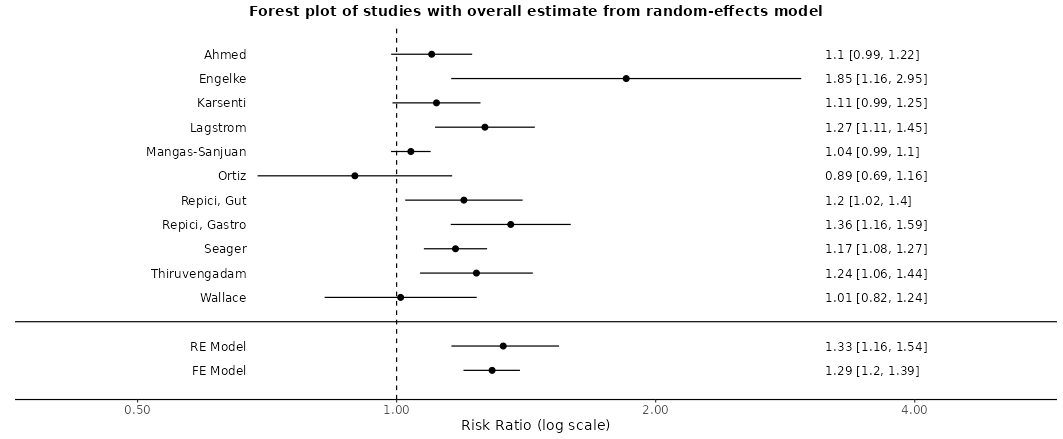


Appendix S10. Forest plots showing the results of sensitivity analyses evaluating the robustness of the primary network meta-analysis. Risk ratios (RRs) with 95% credible intervals (CrIs) are shown, estimated using a Bayesian random-effects network meta-analysis. Values greater than 1 indicate higher ADR compared with control.

(a) Studies with no statistically significant difference in withdrawal time between groups.


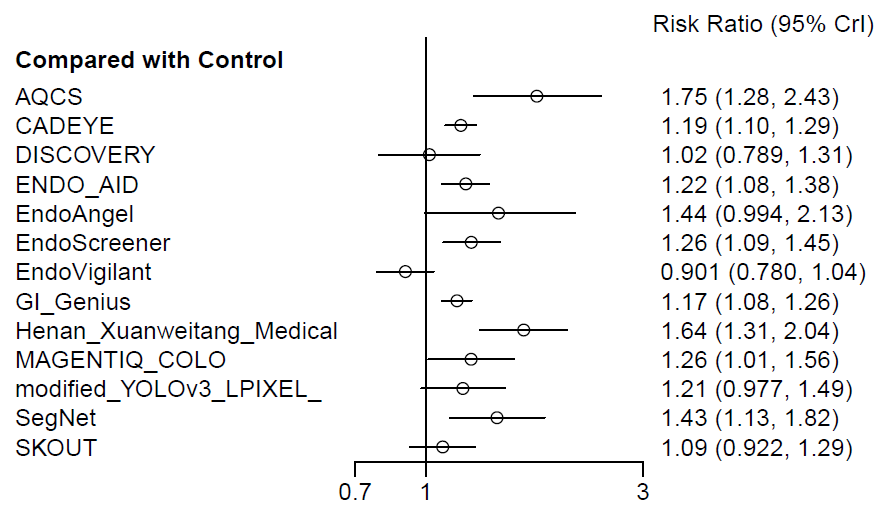


(b) Studies without conflicts of interest related to CADe manufacturers.


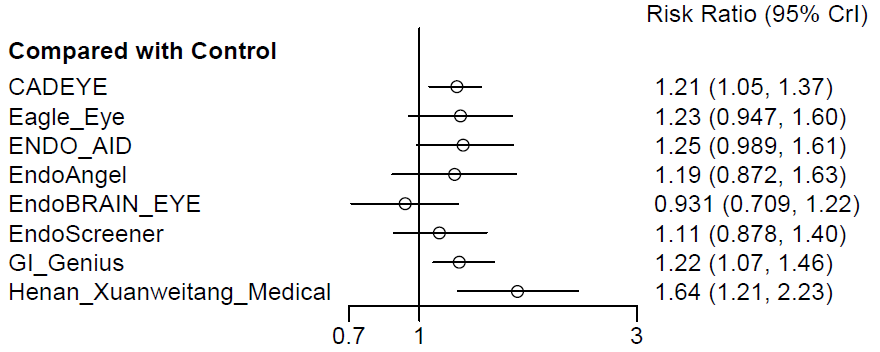


(c) CADe systems evaluated in three or more randomized controlled trials.


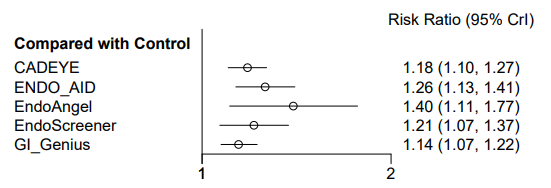


(d) Exclusion of the single study (Miyaguchi et al) in which CADe was used in combination with image-enhanced endoscopy.


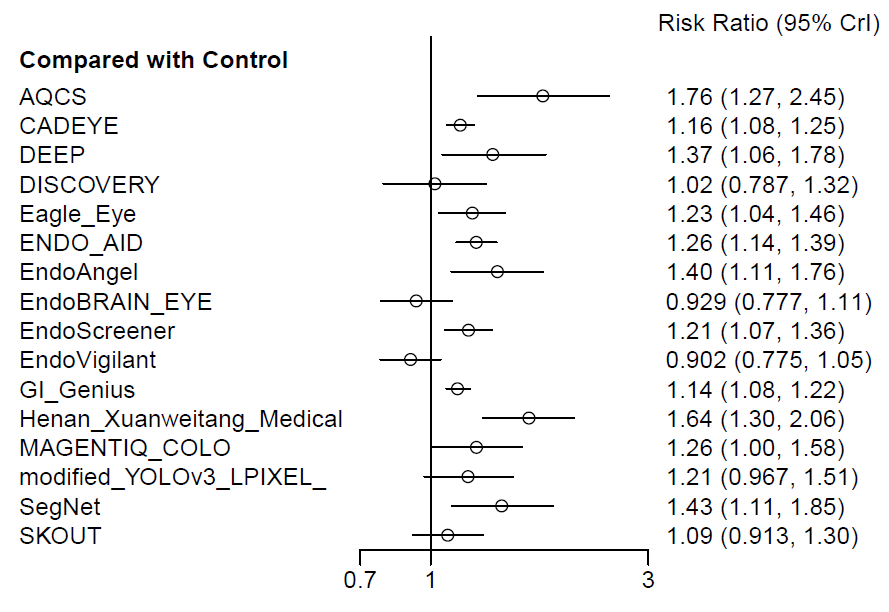


(e) Parallel-group randomized controlled trials only.


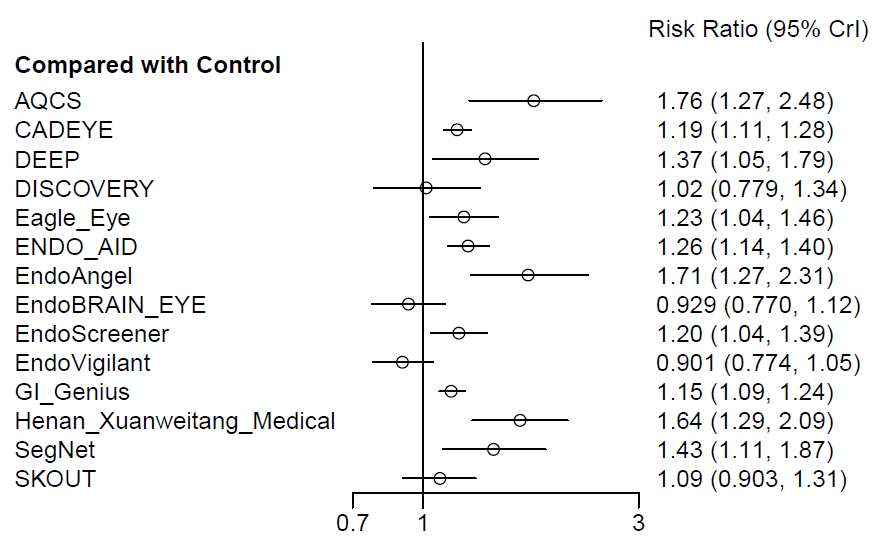


(f) Multicenter randomized controlled trials only.


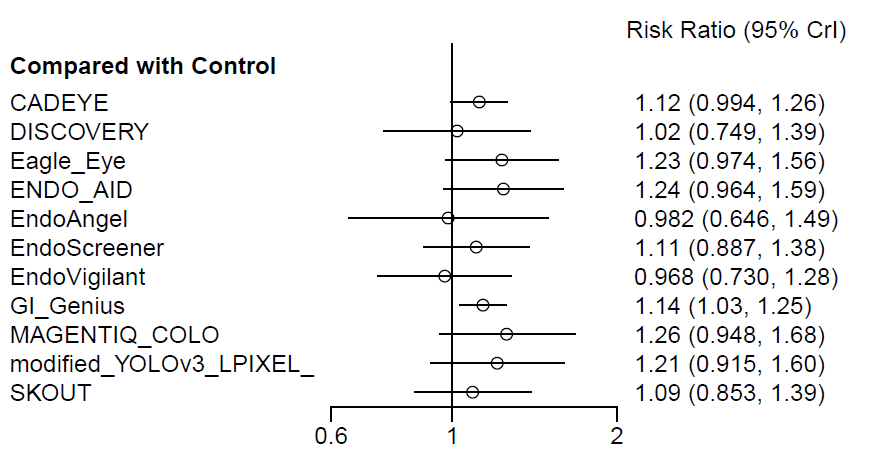


(g) Studies in which colonoscopies were performed exclusively by experienced endoscopists.


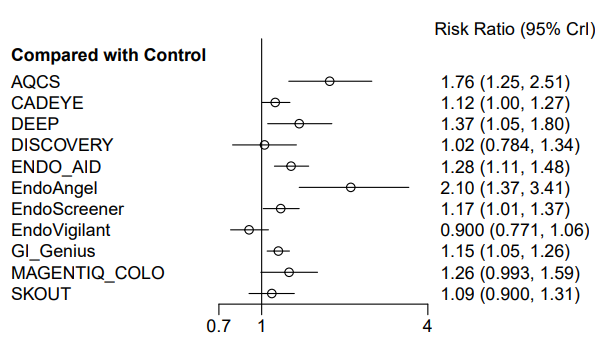


(h) Studies published between 2023 and 2025.


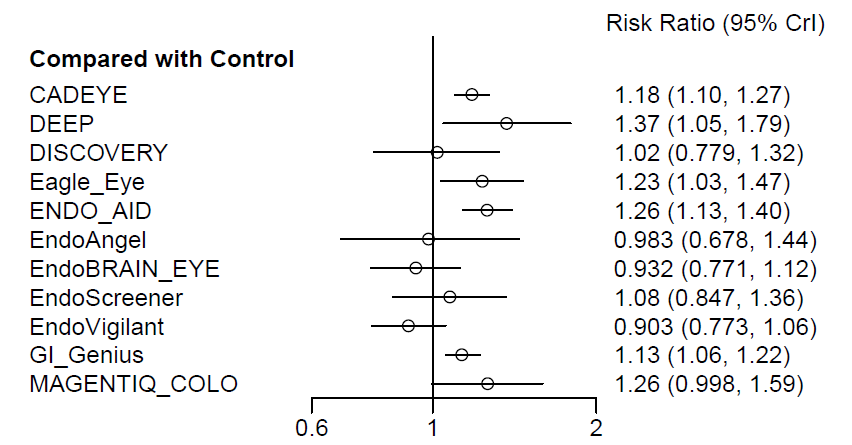


Appendix S11. Withdrawal time in the included studies.

| First author |  | CADe | Control | p-value |
| --- | --- | --- | --- | --- |
| *Alali | Mean, sec | 542 | 509 | 0.15 |
| *Aniwan | Median, min | 9.5 | 9.2 | 0.08 |
| *Desai | Mean, min | 11.2 | 10.8 | 0.11 |
| Djinbachian |  | NR | NR | NR |
| Hiratsuka | Median, min | 14.7 | 14.3 | NR |
| *Hüneburg | Mean, min | 15 | 14 | 0.17 |
| *Miyaguchi | Mean, min | 7 | 7 | 0.96 |
| *Nakashima | Mean, sec | 411 | 399 | 0.20 |
| *Rondonotti | Median, sec | 540 | 540 | 0.4 |
| Tiankanon | Median, min | 10 | 9 | NR |
| *Yamaguchi | Mean, min | 13 | 13 | 0.97 |
| Zimmermann-Fraedrich | Median, sec | 520 | 494 | NR |
| *Ahmad | Median, min | 14.9 | 13.9 | 0.34 |
| Engelke | Median, min | 11 | 10 | 0.03 |
| Karsenti | Median, sec | 480 | 435 | 0.001 |
| Lagström |  | NR | NR | NR |
| Mangas-Sanjuan | Mean, min | 10.6 | 9.8 | <0.05 |
| *Ortiz | Median, min | 13.1 | 12.6 | 0.32 |
| *Repici, Gut | Mean, min | 8.1 | 7.9 | 0.17 |
| *Repici, Gastro | Median, min | 7.3 | 7.0 | 0.10 |
| *Seager | Mean, min | 9.8 | 9.3 | 0.11 |
| *Thiruvengadam | Median, min | 7.5 | 7.3 | 0.84 |
| Wallace | Mean, min | 9.9 | 9.5 | NR |
| *Glissen Brown | Median, min | 8.4 | 7.3 | 0.06 |
| *Liu, Ther Adv | Mean, min | 6.4 | 6.5 | 0.24 |
| *Wang, Gastro | Median, min | 6.5 | 6.5 | 0.74 |
| *Wang, Lancet | Mean, min | 6.4 | 6.3 | 0.14 |
| Wang, GR | Mean, min | 8.2 | 7.6 | <0.001 |
| *Gimeno-García | Mean, min | 7.9 | 7.8 | 0.29 |
| *Lui | Mean, min | 9.3 | 8.4 | 0.18 |
| Lau | Mean, min | 14.9 | 13.7 | 0.04 |
| *Spada | Mean, min | 9.8 | 9.9 | 0.96 |
| Vilkoite |  | NR | NR | NR |
| Gong | Mean, min | 6.3 | 4.7 | <0.001 |
| *Yao, Endosc | Mean, min | 10.5 | 9.7 | 0.056 |
| Yao, GIE | Mean, min | 7.3 | 7.2 | NR |
| *Wei, AJG | Mean, min | 8.8 | 9.1 | 0.289 |
| *Wei, AIMI | Mean, min | 22 | 22.5 | 0.755 |
| *Kamba | Mean, sec | 434 | 449 | 0.104 |
| Lachter | Mean, min | 7.2 | 6.6 | NR |
| *Liu, Saudi | Mean, min | 6.37 | 6.32 | 0.52 |
| *Maas, Endosc | Median, min | 9.2 | 9.0 | 0.05 |
| *Shaukat | Mean, min | 8.91 | 8.43 | 0.072 |
| *Su | Mean, min | 5.41 | 5.54 | 0.355 |
| *Wang, Gut | Mean, min | 6.17 | 6.13 | 0.15 |
| Xu | Median, min | 8.25 | 7.78 | 0.004 |
| *Maas, Lancet | Median, min | 6.5 | 6.5 | 0.86 |
| Yabuuchi | Mean, sec | 751 | 691 | 0.034 |

NR: not reported

* Included studies (n = 31) in the sensitivity analysis regarding withdrawal time

Appendix S12. Disclosure of conflicts of interest (COI) related to manufacturers of CADe devices used in the included studies

| First author | System | Company | COI |
| --- | --- | --- | --- |
| *Alali | CADEYE | Fujifilm | Absent |
| *Aniwan | CADEYE | Fujifilm | Absent |
| Desai | CADEYE | Fujifilm | Present |
| Djinbachian | CADEYE | Fujifilm | Present |
| *Hiratsuka | CADEYE | Fujifilm | Absent |
| Hüneburg | CADEYE | Fujifilm | Present |
| *Miyaguchi | CADEYE | Fujifilm | Absent |
| Nakashima | CADEYE | Fujifilm | Present |
| Rondonotti | CADEYE | Fujifilm | Present |
| *Tiankanon | CADEYE | Fujifilm | Absent |
| *Yamaguchi | CADEYE | Fujifilm | Absent |
| Zimmermann-Fraedrich | CADEYE | Fujifilm | Present |
| *Ahmad | GI Genius | Medtronics | Absent |
| *Engelke | GI Genius | Medtronics | Absent |
| Karsenti | GI Genius | Medtronics | Present |
| *Lagström | GI Genius | Medtronics | Absent |
| Mangas-Sanjuan | GI Genius | Medtronics | Present |
| Ortiz | GI Genius | Medtronics | Present |
| Repici, Gut | GI Genius | Medtronics | Present |
| Repici, Gastro | GI Genius | Medtronics | Present |
| Seager | GI Genius | Medtronics | Present |
| *Thiruvengadam | GI Genius | Medtronics | Absent |
| Wallace | GI Genius | Medtronics | Present |
| *Glissen Brown | EndoScreener | Shanghai Wision | Absent |
| Liu, Ther Adv | EndoScreener | Shanghai Wision | Present |
| Wang, Gastro | EndoScreener | Shanghai Wision | Present |
| Wang, Lancet | EndoScreener | Shanghai Wision | Present |
| *Wang, GR | EndoScreener | Shanghai Wision | Absent |
| Gimeno-García | ENDO-AID | Olympus | Present |
| *Lui | ENDO-AID | Olympus | Absent |
| Lau | ENDO-AID | Olympus | Present |
| Spada | ENDO-AID | Olympus | Present |
| *Vilkoite | ENDO-AID | Olympus | Absent |
| Gong | EndoAngel | Wuhan ENDOANGEL Medical Technology | Present |
| *Yao, Endosc | EndoAngel | Wuhan ENDOANGEL Medical Technology | Absent |
| *Yao, GIE | EndoAngel | Wuhan ENDOANGEL Medical Technology | Absent |
| Wei, AJG | EndoVigilant | EndoVigilant | Present |
| Wei, AIMI | EndoVigilant | EndoVigilant | Present |
| Kamba | modified YOLOv3 (LPIXEL) | LPIXEL | Present |
| Lachter | DEEP | Verily Life Sciences LLC | Present |
| *Liu, Saudi | Henan Xuanweitang Medical | Henan Xuanweitang Medical Information Technology | Absent |
| Maas, Endosc | DISCOVERY | PENTAX Medical | Present |
| Shaukat | SKOUT | Iterative Scopes | Present |
| Su | AQCS | Wuhan EndoAngel Medical Technology | Present |
| Wang, Gut | SegNet | Shanghai Wision | Present |
| *Xu | Eagle-Eye | Xiamen Innovision Technology | Absent |
| Maas, Lancet | MAGENTIQ-COLO | Magentiq Eye | Present |
| *Yabuuchi | EndoBRAIN-EYE | Cybernet systems | Absent |

* Included studies (n = 19) in the sensitivity analysis regarding COI
